# Stakeholder Attitudes towards Donating and Utilizing Donated Human Breastmilk

**DOI:** 10.3390/ijerph16101838

**Published:** 2019-05-23

**Authors:** Welma Lubbe, Charlene S. Oosthuizen, Robin C. Dolman, Namukolo Covic

**Affiliations:** 1 Quality in Nursing and Midwifery (NuMIQ) research unit, School of Nursing Science, North-West University, Potchefstroom 2520, South Africa; 2 Centre of Excellence for Nutrition, North-West University, Potchefstroom 2520, South Africa; Charleneoost@gmail.com (C.S.O.); Robin.dolman@nwu.ac.za (R.C.D.); n.covic@cgiar.org (N.C.)

**Keywords:** breastfeeding, donated human breast milk, breast milk bank, attitudes, acceptability

## Abstract

The promotion and support of human milk banks (HMBs) can enhance exclusive breastfeeding rates. The success and sustainability of HMBs depend on the support from relevant healthcare workers and related communities. This study aimed to determine attitudes of key stakeholders, including mothers, healthcare workers and grandmothers, regarding the donation and receipt of human breastmilk. This study was conducted at a public hospital and clinics in the North West Province, South Africa. Eight focus group discussions explored the attitudes regarding donating and receiving human breastmilk: three groups with mothers of infants (n = 13), three with grandmothers (>60 years old) (n = 17) and two with healthcare professionals working with infants (n = 11). Four main themes emerged: perception regarding breast and formula feeding; exposure to the concept of “wet nursing”; breastmilk donation; and utilization and opinions of community members and traditional healers. Specific barriers identified included the processes for donating and receiving milk, safety, human immuno-deficiency virus (HIV) screening and cultural beliefs. Mothers’ fears included having insufficient milk for their own infants, changes in the quality of donated milk during pasteurization and transportation and HIV transmission. Despite barriers towards donations to and the use of HMBs, sufficient information could enhance donations by mothers and breastmilk utilization.

## 1. Introduction

The benefits of exclusive breastfeeding (EBF) on the growth, development and health of infants are widely known. Numerous studies have reported improved maternal and infant health outcomes [1,2,3,4]. Breastmilk provides the unique balance of nutrients to meet the nutritional needs of growing infants [1]. The Bellagio Child Survival Study Group identified breastfeeding during the first year as an important strategy for improving child survival [5,6]. Moreover, according to a United Nations Children’s Fund (UNICEF) report (2012), EBF is the most effective global intervention for reducing the risk of neonatal infections such as pneumonia and diarrhea, with a significant effect on the reduction of mortality compared to non-EBF [7].

Breastmilk is the optimal food for preterm infants. Benefits include protection against infections, providing all the nutrients needed for growth and protecting the infant against necrotizing enterocolitis (NEC) [8,9,10], a common gastrointestinal emergency among newborn infants [11,12,13]. NEC is an inflammatory gastrointestinal (GI) disease process characterized by tissue necrosis and multisystem organ failure, causing an acute clinical presentation of feeding intolerance, bloody stools, cardiorespiratory compromise and severe hemodynamic instability [10,11,12,13,14]. Both the mother’s own milk and donor human milk have been associated with significantly reduced incidences of NEC [9,14,15,16,17,18,19].

Therefore, donor milk is the best option in the absence of a mother’s own milk. Human milk banks (HMBs) play an important role in the promotion of breastfeeding. An increased rate of breastfeeding at discharge from hospital was observed for very low birth weight (VLBW) infants when donated breastmilk had been provided in the NICU [20]. Due to the supporting evidence, South Africa declared, in the Tshwane declaration of 2011, that HMBs should be promoted and supported as effective approaches to promoting breastmilk feeding. Horton, et al. [21] measured the costs and impact of three breastfeeding promotion programs in developing countries [22,23,24] and reported that breastfeeding and HMBs were amongst the most cost-effective preventive health interventions [21,22,23,24].

Numerous factors determine the success and sustainability of an HMB, including the support and commitment of the relevant healthcare workers, as well as the relevant community. It is therefore vital to determine the attitudes of the community, as well as those of the healthcare workers, towards HMBs. Understanding the attitudes of doctors, nurses and dieticians who work with mothers, could provide valuable information for enhancements of future training programs and policies regarding the start-up of HMBs. In addition, understanding the attitudes of various community members served by the HMB could enable the Department of Health and healthcare workers to develop appropriate educational materials and messages for these communities and other stakeholders. In South African communities, research has also highlighted the involvement and influence of family members, and especially grandmothers, in a community as an important factor influencing infant feeding choices, and they are therefore also important stakeholders [25,26,27,28].

South Africa has a low breastfeeding rate of only 8% for infants younger than six months, with the lowest exclusive breastfeeding (EBF) rate in the North West Province, despite evidence supporting EBF [29] and an active HMB in this province. Limited South African research is available about the acceptability and attitudes of stakeholders toward HMB, especially in the North West Province. This study therefore aimed to determine the attitudes of stakeholders (including mothers, grandmothers and healthcare professionals) towards donating and using donated human breastmilk. 

## 2. Materials and Methods 

This study was performed in the North West Province of South Africa. The researchers used an observational approach and conducted focus group discussions (FGDs) with key informant participant groups to identify their attitudes about donating and using donated breastmilk. 

Three groups of stakeholders were included for participation in the FGDs: mothers (M) with infants aged 0 to 12 months; grandmothers (G) in the community (at least 60 years old); and healthcare professionals (HC) who cared for mothers with infants aged 0–12 months regularly, who operated in healthcare facilities. Each group was purposely selected due to the potential influence on mothers’ choices regarding breastmilk donation. Most participating mothers were 25–34 years old, most grandmothers were 55–64 years old and most healthcare professionals were 25–34 years old. The mothers’ education levels ranged from grade 12 (n = 3) to grade 4 (n = 1). Only 35% (n = 6) of the grandmothers had acquired some education. Most participants were unemployed, except in the healthcare professional group, where all participants were employed. 

Data were collected at two district clinics and one hospital in the Kenneth Kaunda district from April to June 2014. The hospital was awarded Mother and Baby Friendly Health Initiative (MBFHI) status, while the two clinics have obstetric facilities. Approximately 529 infants visited these facilities per month; therefore, the specific catchment population could sustain the established HMB. 

A trained research assistant facilitated the FGDs conducted with the mothers and the grandmothers in the local language, Setswana. The principal investigator completed two practice sessions with the research assistant to ensure that she became familiar with the procedure. The research assistant also explained the study’s aim, inclusion and exclusion criteria, HMB procedures, and obtained informed consent. She approached participants whilst waiting in queues at selected clinics on specific infant consultation days. Special arrangements were made with the clinic and hospital management for mothers who participated in the FGDs not to lose their places in queues. 

The principal investigator conducted two FGDs with the healthcare professionals, ensuring that it did not interfere with their working schedules. In total, eight FGDs were conducted: three with mothers (n = 13), three with grandmothers (n = 17) and two with healthcare professionals (n = 11). Privacy and anonymity were ensured by conducting the discussions in a separate room and omitting the names of the participants from any discussions. Each participant received a number during transcription according to the order in which they participated—for example, P1 and P2—ensuring that specific participants could not be identified. 

The sessions commenced by providing information about the research project, obtaining informed consent and collecting demographic data from the participants. The FGD guide consisted of 13 open-ended questions for mothers and grandmothers and nine open-ended questions for healthcare professionals (Table 1). These were adapted from a similar study by Coutsoudis et al. with these authors’ permission [28]. The researchers encouraged robust discussion during all the FGDs. Data saturation was reached in each FGD.

### 2.1. Focus Group Discussions and Analysis

Eight FGDs were conducted involving 41 participants. All participants lived in the catchment area of the HMB. All FGDs were audio recorded and transcribed verbatim in Setswana and then translated into English by the research assistant. A second research assistant also transcribed and translated the transcripts. Both transcriptions were compared for any discrepancies but none were found. To ensure anonymity, all digital audio recordings were stored on a password-protected computer and permanently deleted from the recording devices.

The transcriptions were analyzed and coded, considering both manifest and latent content to determine themes and sub-themes of the types of attitudes that emerged. Quotes by mothers are indicated as M, grandmothers as G and healthcare professionals as HC. 

Specific categories of attitudes were analyzed and the principal investigator did the coding twice, once by hand using a qualitative content analysis approach and once using the software package Atlas. Ti, version 7.1.8 (Scientific Software Development. GmbH, Berlin, Germany). A co-coder also coded the transcriptions independently to enhance rigor. The principal investigator and co-coder jointly compared and finalized the themes and labelled them.

### 2.2. Ethical Considerations

Ethical approval was obtained from the North-West University Health Research Ethics Committee (number NWU-00083-13-S1). Permission to undertake the study was obtained from the North West Department of Health and the Tlokwe sub-district office in the Dr Kenneth Kaunda district. Approval to collect data in specified clinics was granted by the district primary health care (PHC) manager and by the managers of the selected healthcare facilities. Written voluntary consent was obtained from every participant after the purpose of the discussion had been explained for the use of their statements from the FGDs and permission to audio record the discussions. Consent forms were available in the three local languages to ensure that each participant could choose his/her preferred language. Confidentiality was assured since no identification could be made from specific statements. 

## 3. Results

Most participating mothers were 25–34 years old, most grandmothers were 55–64 years old and most healthcare professionals were 25–34 years old. The mothers’ education levels ranged from grade 12 (n = 3) to grade 4 (n = 1). Only 35% (n = 6) of the grandmothers had acquired some education. Most participants were unemployed, except in the healthcare professional group, where all participants were employed. Quotes by mothers are indicated as M, grandmothers as G and healthcare professionals as HC, followed by the participant’s number and the number of the statement for the said participant.

Four main themes were identified from the analyzed FGD data, namely (1) perceptions regarding breastfeeding and formula feeding; (2) exposure to the concept of wet nursing; (3) breastmilk donation and utilization; and (4) opinions of community members and traditional healers. 

### 3.1. Perceptions Regarding Breastfeeding and Formula Feeding

Participants regarded breastfeeding as superior to formula feeding, due to the benefits of breastfeeding. All participants stated benefits, including convenience, since breastmilk is readily available and does not require temperature adjustments or mixing, protection from infections, bonding facilitation and affordability. Mother and healthcare participants stated that formula milk is so expensive that formula might be diluted to prolong its use, which could lead to the early introduction of solids.

“Sometimes the milk is not enough; you then decide to reduce the scale to save some for the following day.” (M3:3)

“They start to give infants food when they are three months because there is no money for formula.” (HP5:1)

Mothers stated that healthcare professionals influenced their decisions to breastfeed, since breastfeeding was encouraged at the local healthcare facilities. Nurses felt that the use of formula was the main reason for malnutrition at their facilities, due to the financial implications of formula feeding. Despite the fact that participants regarded breastfeeding as being the superior option, one mother and one healthcare professional would give formula if they could afford to do so. According to grandmothers, formula feeding whilst at work during the day and breastfeeding at night was a common practice. 

Mothers and grandmothers perceived breastfeeding to occur less frequently, especially among younger mothers. One mother participant stated:

“Usually young mothers live fast lives. It is not usual to find them breastfeeding. It is only older woman who do it. The young women are after the fun times.” (M2:1)

Some reasons provided for the decreased prevalence of breastfeeding included that breastfeeding is no longer trendy among peers, peer pressure favored formula feeding, that it is impractical for mothers returning to work, mothers’ beliefs that they have insufficient amounts of breastmilk and the local cultural belief that mothers who breastfeed are not allowed to have sexual intercourse whilst breastfeeding: 

“They have boyfriends so they just want to get back to them, like according to our culture you should mourn in a way of respect for your child when you breastfeed, for about 3–4 months without sex, so young mothers think four months is too much. They can’t wait for 4 months, so it’s best for them to bottle-feed so that they have sex freely.” (M1:1)

### 3.2. Exposure to the Concept of Wet Nursing

Some participants knew about wet nursing, through direct family experiences or having practiced it themselves. One grandmother remarked:

“I know it, I have done it myself but in the family. My cousin and I were both breastfeeding. When she was not home, I would feed her baby my milk as the baby was only fed breastmilk.” (G2:1)

However, a number of participants, especially in the grandmother participant category, no longer condoned wet nursing due to fears of diseases that might infect the babies, especially HIV. Mothers remarked about checking their “status”. However, participants indicated that they would be willing to practice wet nursing if they knew the other person involved, and knew her status; they added that it had to be a family member to ensure that they were comfortable with this practice. 

The discussion about wet nursing revealed the fear that the infant would bond with the wet nurse. This fear also surfaced during discussions about fears regarding the donation and utilization of donor human milk. Participants from all three categories were afraid that the infant would bond with the person whose milk he or she drank, or inherit some of the donor’s personality traits when drinking her breastmilk:

“You might lose the love from mother to child.” (M3:2)

“I breastfeed the child or if it is my child I give the child to someone to feed the child; there is something we call a bond when you breastfeed. So, that means that person will bond with my child.” (M3:1)

### 3.3. Awareness of Human Milk Banking

All participants had heard about human milk banking, mostly from healthcare facilities. However, not everyone knew which processes were followed during milk banking, except for healthcare participants who were familiar with these processes. 

“But if you understand more, I don’t think there will be any problems. If nurses in clinics can explain it well, like you have just done, people will understand more and know how important it is to donate.” (M1:D5)

### 3.4. Attitudes and Perceptions Regarding the Donation of Breastmilk

Participants were asked how they felt about donating breastmilk and what difficulties donors might encounter. Mother and healthcare participants raised questions concerning whether donation would affect the availability of breastmilk for their own children, cause the donor to feel weak or exhausted, result in fluctuation of breast size, and about the effort required to express milk for donation:

“To express, it’s difficult sitting the whole day expressing.” (M4:5)

These questions did not arise during the grandmothers’ discussions.

As part of the discussion process, participants asked for clarification about what happened to the donated milk and how donors were screened. After these explanations, participants were more receptive about the idea of breastmilk donation. However, one mother remained concerned about the reaction that HIV testing would invoke from potential donors, and commented:

“Some people don’t like to go through tests. They ask themselves, what if they are ill; those are the fears people have.” (M4:5)

This was, however, an isolated concern, as all groups discussed that proper safety testing would make them feel more at ease about human milk banking. 

### 3.5. Attitudes and Perceptions Regarding Receiving Donated Breastmilk

When participants were asked how they felt about their own infants or grandchildren receiving donated breastmilk, they again discussed safety as a main concern, especially regarding HIV transmission. They indicated that screening tests had to be implemented to ensure that the donor is healthy. One mother reported:

“If they have checked my milk and that of another woman, they are the same as long as they are clean.” (M4:1)

A final issue was related to who the donor was. When addressing this issue with the healthcare participants, one felt that it would only be acceptable if the donor was a relative. There seemed to be sensitivity towards the issue of race among the participant categories, as they felt that it mattered who the donor was and from which culture they came, as is evident from the statement of one mother:

“We black people have a tendency of saying English is not my language, as it is something you never got from your mother. So, if a child drinks someone else’s’ breastmilk, they will be confused.” (M3:1)

“Some families have heritages, so I would be giving another child my heritage.” (HC3:1)

“Let’s say you are a slow learner; that baby can also be a slow learner too.” (HC2:1)

Healthcare participants suggested that screening should involve testing heritage and genes to ensure that the cultures were compatible, although participants also felt that if donor breastmilk has been tested for diseases and safety, it would be sufficient.

Healthcare staff members raised concerns about practical issues, such as the transport of the donated milk from the donor to the bank and the logistics, including maintaining the cold chain and the effects on donor milk quality, as communicated by one of the doctors:

“Now it’s transported to the clinics, with refrigeration of the milk, you don’t know the quality of the milk when it reaches you.” (HC5:2)

### 3.6. Perceived Opinions of Community Leaders and Traditional Healers

When asked about perceptions of community leaders and traditional healers about the donation and utilization of donor human breastmilk, the participants had mixed responses, as some felt that community leaders and traditional healers would support the venture if they received proper education, while others disagreed. A summary of the suggestions resulting from the FGD is provided in Table 2.

## 4. Discussion

It was important to determine participants’ perceptions regarding breastfeeding versus formula feeding, as these perceptions indicate where education should be initiated. Most participants stated that breastfeeding was superior to formula feeding, mainly because of the cost of formula feeds, benefits for the infant’s health, convenience, strengthening of bonding between mother and child and risks associated with formula feeding. Other studies support these findings [26,27,30].

Although the participants supported breastfeeding, the reviewed literature indicates that younger women had significantly lower rates of breastfeeding than older women [27,31,32]. Grandmothers and nurses stated that the prevalence of breastfeeding has decreased and that few modern mothers in developing countries practiced EBF.

Early education about feeding options is important, as the literature suggests that mothers decide on their feeding option before and during early pregnancy [30,33]. In the current study, mothers also indicated that their infant feeding choices were influenced by the health education they received during pregnancy. Furthermore, mothers who breastfeed were more prone to support HBMs and felt that breastmilk provided the optimum nutrition for infants [34]. During the discussion with healthcare professionals, a few negative perceptions arose, such as concerns about the quality of donor milk after transportation and issues related to the sources of donor milk, as they felt the genes and heritage of the donors were important. As education prior to pregnancy and during early pregnancy is mainly the responsibility of healthcare staff, it is evident that sufficient attention has been paid to the training of healthcare staff on such issues. O’Sullivan, et al. [35] found that healthcare workers can play a part in facilitating the donation of milk and that they have the responsibility to inform the mother of all her options when it comes to infant feeding. In a recent study in Indonesia, the acceptability of DBM was, however, still low among healthcare workers and mothers [36]. The participants in the current study were divided on whether traditional healers and community leaders would support HBMs or not. Traditional healers and community leaders should also be specifically targeted regarding training about infant feeding-related issues, as they might influence the wider community as their opinions are highly valued.

Strong cultural beliefs might influence mothers’ choices of feeding, such as the belief that sexual intercourse should be avoided whilst breastfeeding. This tendency was mentioned in two other studies performed in Africa (Tanzania [37] and Cameroon [38]), but no such report in South Africa could be found. Such a belief would encourage mothers to cease breastfeeding early. This concern might pose a barrier to EBF in South Africa, the prevalence of which needs to be explored in specific communities. Participants also raised cultural concerns about using donated human milk, since they feared that certain personality traits could be transferred from the donor to the recipient infant.

These concerns demonstrated the need for education early during pregnancy to ensure mothers receive the correct scientific facts about infant feeding. Health education concerning this topic should also include grandmothers, as culture, perceptions and beliefs are transferred from generation to generation. Furthermore, community leaders and traditional leaders should also be involved as they influence communities’ decisions and perceptions. 

The mother and grandmother informants included participants who had experienced or practiced wet nursing or the informal sharing of breastmilk themselves. Individuals who have experienced wet nursing positively might be more positive towards human milk donation. Participants in the FGDs who had previously practiced wet nursing were unsure whether they would do so again; they had concerns about safety issues, especially the risk of HIV transmission in expressed human milk. A previous study performed in South Africa reported similar findings [28], as did a study in New York [35]. These findings emphasize the need for education, especially on breastfeeding in the context of HIV and the safety precautions taken in HMB operations. 

Focus group participants in all three categories knew about HMB, although some were unsure about the criteria and processes involved. This was expected, as only one HMB in the province had been opened and one facility had achieved MBFI accreditation during the course of the study—the first in the province. Some mothers were concerned that the donation of breastmilk may affect their health. Some mothers might be discouraged from donating breastmilk because HIV screening tests were required. The participants recommended that the acceptability of donating and receiving donor human milk could be enhanced if the procedures, benefits and processes would be broadcasted effectively throughout the community to attract potential donors and users. Pamphlets, radio talks and community meetings might be a productive method for reducing skepticism regarding HMBs in South Africa. A participant commented that education would help to make human breastmilk banking more acceptable among stakeholders. 

### Limitations

The limitations of this study include the fact that the perceptions, beliefs and opinions presented by the informants cannot be generalized due to the qualitative nature in which they were observed. However, the information obtained could form a basis upon which to design a structured questionnaire that could be used to determine the prevalence of the observed perceptions, beliefs and opinions among the wider populations of stakeholder categories.

## 5. Conclusions

The study shed light on possible barriers affecting the acceptance or donation of human breastmilk from HMBs. These barriers include the processes of donating and receiving milk, including testing for HIV, cultural beliefs relating to the transfer of personality traits and bonding, fears of having insufficient milk for their own babies, fears that the expressed milk might cause ill health, fears of changes in the quality of donated milk during pasteurization and transportation and fears of HIV transmission. In addition, possible barriers to exclusive breastfeeding were identified, such as the cultural requirement to avoid sexual intercourse while breastfeeding, peer pressure and breastfeeding being unfashionable. Given these findings, the researchers recommend further research to determine the prevalence of the identified beliefs, opinions and attitudes in communities served by HMBs. The identified factors from this study could form the basis for designing an appropriate structured questionnaire. More research is required about the opinions and attitudes of community leaders and traditional healers regarding HMB.

## Figures and Tables

**Table 1:** Open-ended questions used during focus group discussions with mothers and grandmothers, as well as healthcare professionals.

FGD Guidelines for Informant Mothers and Grandmothers
1. Between formula and breast milk, which do you feel is the better choice for feeding infants and young children, and why?
2. What do you think the mothers should use to feed their babies?
3. How common do you think breastfeeding is in your community?
4. Has anyone heard of something called ‘wet nursing,’ or the practice of giving a child, whose mother cannot breastfeed, to another mother to breastfeed the infant/child for her?
5. What do you think about this practice? And have you ever heard of it being practiced before?
6. Has anyone heard of something called ‘breast milk donation? If so: Could you explain what it is?
7. What do you think about this practice of breast milk donation?
8. Do you think most women would be willing to donate their milk?
9. Would you be willing to donate your milk if you were breastfeeding?
10. What might prevent women from wanting to donate their milk?
11. What will traditional healers and community leaders think of breastmilk donation?
12. Do you have (or have you heard of) any fears about donating your milk?
13. What do you think will help promote breastmilk donation?
**FGD Guideline for Healthcare Informants (Additional Questions)**
1. What are your thoughts on the establishment and operation of the HMB at the local hospital?
2. Have you been involved at all with this HMB?
3. Is there anything you would like to see done differently at the HMB?
4. How can we make it easier for you to prescribe/supply donor breastmilk?

FGD: focus group discussions. HMB: human milk bank.

**Table 2:** Suggestions made by focus group participants for improving the acceptability of human donor breast milk.

Suggestions for Improving the Acceptability of Human Donor Milk	
Mothers and Representatives of Grandmothers	Healthcare Professionals
Improving general knowledge about human milk banking; for example: How it works Why it is important Inclusions and exclusions	Improving general knowledge about human milk banking; for example: How it works Why it is important Inclusions and exclusions
Advertising: Television Radio talks Pamphlets Health education talks	Advertising: Television Radio talks Pamphlets Health education talks
	Rewarding of donors
	Testing for heritage and genes
	Setting guidelines to ensure that breastmilk is not misused or abused
	Continuation of recruiting donors
	Increasing awareness of human milk banking among doctors and paediatricians
	Monthly follow up contacts with donor
	Criteria specifying who may receive donated breastmilk
